# Book Reviews

**Published:** 1908-08

**Authors:** 


### BOOK REVIEWS

---

PROGRESSIVE MEDICINE. A Quarterly Digest of Advances, Discoveries, and Improvements in the Medical and Surgical Sciences. Edited by H. A. Hare, M. D., Assisted by H. R. M. Landis, M. D. Vol. II, June, 1908. Published by Lea & Febiger, Philadelphia.

This volume of Progressive Medicine, well sustains the reputation of this valuable quarterly. The subject of Hernia is thoroughly discussed by William B. Coley, who describes the different improvements and variations in the management of this condition. The other abdominal surgical procedures are taken up by E. M. Foote, who devotes 80 pages to this subject and reviews the recent literature. Jno. G. Clark covers the review of Gynecology in 102 pages and presents the "kernel" of the literature of the past year. Diseases of the Blood, Diathetic and Metabolic Diseases, Diseases of the Spleen, Thyroid Gland and Lymphatic system are next taken up by Alfred Stengel. The subject of Ophtramology by Edward Jackson, completes this volume.

To obtain quickly a brief resume of the year's work in any one of these fields of medicine we know of no more valuable work, and commend most highly Progressive Medicine as a conservative "up-to-date" quarterly.

A MANUAL OF PERSONAL HYGIENE: Proper Living upon a Physiologic Basis. By Eminent Specialists. Edited by Walter L. Pyle, M. D., Assistant Surgeon to the Wills Eye Hospital, Philadelphia. Third Revised Edition. 12mo of 451 pages, illustrated. Philadelphia and London: W. B. Saunders Company, 1907. Cloth, $1.50 net.

W. B. Saunders Company, Philadelphia and London.

The object of this manual is to set forth plainly the best means of developing and maintaining physical and mental vigor. It represents a thorough exposition of living upon a physiologic basis. There are chapters upon the hygiene of the digestive apparatus, the skin and its appendages, the vocal and respiratory apparatus, eye, ear, brain, and nervous system.

In the new third edition just issued, the work has been thoroughly revised and numerous additions have been made, including an illustrated System of Home Gymnastics, a chapter on Domestic Hygiene, and an Appendix containing the simpler methods of hydrovteraphy, thermotheraphy, and emchanopheraphy, and a section of First Aid in Medical and Surgical Accidents and Emergencies.

An urgent need is met by this book, in furnishing a work which physicians may safely put into the hands of their patients without fear of inculcating false impressions.

---

MODERN SURGERY: General and Operative. By J. Chalmers DaCosta, M. D., Professor of the Principles of Surgery and of Clinical Surgery in the Jefferson Medical College, Philadelphia. Fifth Revised Edition, Enlarged and Reset. Octavo volume of 1283 pages, with 872 illustrations, some in colors. Philadelphia and London: W. B. Saunders Company, 1907. Cloth, $5.50 net; Half Morocco, $6.50 net.

W. B. Saunders Company, Philadelphia and London.

For this new fifth edition Dr. DaCosta's Modern Surgery has been practically rewritten. Some of the new subjects added are: Ransohoff's plan of discission of the pleura in chronic empyema; Brophy's operation for cleft palate; scopolamin-morphin anesthesia, local anesthesia by injection of stovain; radium; Willy Meyer's operation for carcinoma of mammary gland; Young's perineal prostatectomy; the Johns-Hopkins operation for inguinal hernia; the Quenu-Mayo operation for rectal cancer; Moynihan's short-loop method of gastrojejunostomy; the no-loop method of gastrojejunostomy, derived by the Mayo brothers; and appendicostomy. Over one hundred and fifty new illustrations have been added.

---

SUBCUTANEOUS HYDROCARBON PROTHESIS. By F. Strange Kolle, M. D. The Grafton Press, Publishers, New York. Price, $2.50 net.

The author of this work has presented a thoroughly practical and concise treatise on the subcutaneous employment of paraffine and vaseline.

Much work has been done on this subject within the last seven years—Gersuny first advocated this method for the correction of deformities about the face, neck and shoulders in 1900. Kolle has reviewed all of the important literature bearing on this work and has thus greatly enhanced the value of his book. The author warns prospective operators against the "beauty cranks," especially those just about to engage in great theatrical ventures, circus performances and very desirable marriages. These individuals are likely to be both undesirable and dangerous patients.

A great mistake is made in the so-called rapid "filling method," without allowing the injections to accommodate themselves and to organize before others are attempted. Especial attention is given to the question of untoward results; and being thus forewarned the operator is forearmed.

Kolle unreservedly recommends the employment of paraffine in the cold or semi-solid form at the mean temperature of about 110 degrees F. He says he has used the cold injection method in 300 nose cases without a single case of sloughing, embolism or death. There are a few typographical errors, among which may be mentioned, "punctuate" for "punctate" on page 16 and "to" instead of "to be" on page 19.

### NEW EDITION OF GRAY'S ANATOMY.

Gray's Anatomy has maintained such a lead in its own field since its original publication, fifty years ago, that it has won the distinction of being the most important work in all medical literature. Hundreds of thousands of copies have started students at the beginning of their course in medicine, have been kept always at hand, and have been carried to their offices after graduation for guidance in the basic facts of medicine and surgery. Such an announcement as a new edition of "Gray" is therefore of primary importance to everyone concerned with medicine, whatever be his stage or station in medical life.

This new edition, soon to appear, is the result of a thorough revision begun two years ago. In this work Professor J. Chalmers Da Costa and Edward Anthony Spitzka, who occupy, respectively, the chairs of Surgery and of Anatomy in the Jefferson Medical College of Philadelphia, have been associated. Dr. Spitzka unites the qualifications of an anatomist of the first rank with those of an artist as well, a rare combination of powers, hence his delineations convey directly to the reader's eye his own exact knowledge of structure. He has rewritten what has heretofore been the most complez and difficult portion of anatomy, the Nerve System, illustrating it with seventy of his own drawings, so that that subject of recently revolutionized development is at once brought to date and simplified. Every other page has been scanned to reflect the latest knowledge.

"Gray" has always been distinguished by the possession of a quality defying analysis and imitation, namely, its teaching power. In this it reflects the towering genius of its author. Henry Gray died young, but left behind him this imperishable evidence of his consummate knowledge of human structure and of the best methods of imparting it to others. Nature rarely creates a Shakespeare, a Napoleon or a Crichton. Until she creates another Gray his work will stand.

No small part of the observed fact that Gray saves a student half his time and effort and doubles the permanence of his knowledge is due to its illustrations. Quantity of pictures can easily be overdone. Teaching quality is difficult to achieve and impossible to imitate. The great series of "Gray" engravings has always been unique in this essential point of teaching quality.

They enable the eye and mind to co-operate, thus focussing the whole of the reader's power on the subject before him. These graphic demonstrations simultaneously convey the terminology of anatomy by reason of the fact that the names of the parts are engraved directly upon them, whereby the nomenclature and also the position, extent and relations of each part are unconsciously and indelibly fixed in the memory. These are the four cardinal points to know about any structure, and they are conveyed by a method unique in "Gray," and one that is as simple as it is effective. Colors are abundantly used to show muscle-attachments, viens, arteries, lymphatics and nerves.

The possessor of the new "Gray" will have the best issue in which this superb book has ever appeared, and from the foregoing description it may be gathered that it will outdistance competitors by a greater interval even than before.

